# Ex vivo tissue slice culture system to measure drug-response rates of hepatic metastatic colorectal cancer

**DOI:** 10.1186/s12885-019-6270-4

**Published:** 2019-11-01

**Authors:** Steve Z. Martin, Daniel C. Wagner, Nina Hörner, David Horst, Hauke Lang, Katrin E. Tagscherer, Wilfried Roth

**Affiliations:** 1 Institute of Pathology, University Medical Center Mainz, Langenbeckstraße 1, 55131 Mainz, Germany; 2 Institute of Pathology, Charité - Universitätsmedizin Berlin, corporate member of Freie Universität Berlin, Humboldt-Universität zu Berlin and Berlin Institute of Health, Campus Charité Mitte, 10117 Berlin, Germany; 3 Department of General Visceral and Transplantation Surgery, University Medical Center Mainz, Langenbeckstraße 1, 55131 Mainz, Germany

**Keywords:** Ex vivo culture, Colorectal liver metastases, CRLM, Predictive biomarker, Predictive test system

## Abstract

**Background:**

The lack of predictive biomarkers or test systems contributes to high failure rates of systemic therapy in metastasized colorectal carcinoma, accounting for a still unfavorable prognosis. Here, we present an ex vivo functional assay to measure drug-response based on a tissue slice culture approach.

**Methods:**

Tumor tissue slices of hepatic metastases of nine patients suffering from colorectal carcinoma were cultivated for 72 h and treated with different concentrations of the clinically relevant drugs Oxaliplatin, Cetuximab and Pembrolizumab. Easy to use, objective and automated analysis routines based on the Halo platform were developed to measure changes in proliferative activity and the morphometric make-up of the tumor. Apoptotic indices were assessed semiquantitatively.

**Results:**

Untreated tumor tissue slices showed high morphological comparability with the original “in vivo”-tumor, preserving proliferation and stromal-tumor interactions. All but one patients showed a dosage dependent susceptibility to treatment with Oxaliplatin, whereas only two patients showed responses to Cetuximab and Pembrolizumab, respectively. Furthermore, we identified possible non-responders to Cetuximab therapy in absence of RAS-mutations.

**Conclusions:**

This is the first time to demonstrate feasibility of the tissue slice culture approach for metastatic tissue of colorectal carcinoma. An automated readout of proliferation and tumor-morphometry allows for quantification of drug susceptibility. This strongly indicates a potential value of this technique as a patient-specific test-system of targeted therapy in metastatic colorectal cancer. Co-clinical trials are needed to customize for clinical application and to define adequate read-out cut-off values.

## Background

Patients with colorectal carcinoma often develop metastases, foremost in the liver [1, 2]. Modern systemic therapeutic strategies include not only platinum-based chemotherapeutics (e.g. FOLFOX), but also novel targeted agents that are directed against a specific characteristic unique to the tumor cells (e.g. antibodies against Epidermal Growth Factor receptor or Programmed cell death ligand 1). Despite numerous promising new drugs, response rates are relatively low, rendering the prognosis of metastasized colorectal carcinoma still unfavourable [2–6]. Adequate stratification is of the utmost importance to select those patients that show a clinical benefit outweighing the side effects of treatment and justifying high costs. Nowadays, this is performed using extensive molecular profiling to identify predictive biomarkers, but clinical practice shows that response to therapy cannot always be reliably predicted using this approach. So far, very few predictive molecular biomarkers have been identified in the context of colorectal carcinoma, the most prominent of which are mutations of KRAS and NRAS that cause irresponsiveness to anti-EGFR antibodies (e.g. Cetuximab) [7–10]. Other factors such as the tumor-stromal interaction; the specific immune landscape and epigenetic factors seem to play a major role in defining its biological behavior that cannot be predicted with molecular profiling alone [11, 12]. A promising technique to overcome this predicament is to measure therapeutic response using an ex vivo functional assay that cultivates a viable sample of the tumor itself. Various 2D monolayer and 3D models have been proposed and their advantages and disadvantages have been compared in a recent review [13]. The tissue slice culture approach shows the best comparability with the original tumor - preserving tumor morphology and microenvironment - while showing a high experimental success rate as well as a short generation time. Here, the non-fixed viable tumor is cut into thin slices and cultured directly for several days. Recently, few research groups have shown that the functional assessment of primary colorectal carcinoma tissue is feasible using this innovative technique [14–16]. However, stratifying patients with metastatic disease into optimal therapy-regiments requires sampling and cultivation of the metastatic tumor tissue.

In this study, we describe a protocol for optimal tissue slice culture of hepatic metastases of colorectal carcinoma and propose an automated, easy to use and objective readout strategy for measuring susceptibility to Oxaliplatin, Cetuximab and Pembrolizumab.

## Methods

### Patients

Nine hepatic metastasectomy specimens of colorectal carcinoma were included in this study. The patients were treated at the Department of General Visceral and Transplantation Surgery of the University Medical Center Mainz between 2017 and 2018. The study was approved by our institution’s ethics committee. Table 1 depicts the patient’s clinical characteristics.

**Table 1:** Patient characteristics

	Patient 1	Patient 2	Patient 3	Patient 4	Patient 5	Patient 6	Patient 7	Patient 8	Patient 9
Initial Diagnosis	June 2018	March 2014	July 2017	February 2017	May 2017	June 2016	May 2013	March 2013	August 2016
Primary									
localization	rectum	rectum	rectum	rectum	caecum	rectum	rectum	rectum	CUP/iCRC
date resection	June 2019	Mar 2014	Oct 2017	June 2017	May 2017	Nov 2016	June 2013	Mar 2013	n.a.
pTNM	pT3 pN1b pM1a (HEP)	ypT2 pN0 cM0	ypT3 pN0 cM0	ypT2 ypN1a cM0	pT3 pN1a cM0	ypT4b ypN1b cM0	pT4b pN1a pM1a(HEP)	pT3 pN0 cM0	n.a.
L/V/Pn	L1, V1, Pn0,	L0, V0, Pn0	L0, V0, Pn0	L0, V0, Pn0	L0, V1, Pn0	L1, V1, Pn1	V0, L1, Pn1	L1, V0, Pn0	n.a.
UICC-stage	IVA	I	II	IIIA	IIIB	IIIC	IVA	IVA	n.a.
Hepatic Metastasis									
date resection	Nov 2018	May 2018	July 2018	Aug 2018	Aug 2018	Sep 2018	Aug 2017	Aug 2017	July 2017
synchronous - 0, metachronous - 1	0	1	1	1	1	1	0	1	n.a.
Molecular biology									
KRAS	WT	mutation G12D	mutation G12A	mutation G13D	WT	WT	WT	WT	WT
NRAS	WT	WT	WT	WT	WT	WT	WT	mutation G13R “c37G > C”	WT
BRAF	WT	WT	WT	WT	WT	WT	WT	WT	WT
MS-stability	MSS	MSS	MSS	MSS	MSS	MSS	MSS	MSS	MSS
Systemic therapy*	yes	yes	yes	yes	no	yes	yes	no	yes
Checkpoint Inhibition									
PDL-1 IC%	50	23	13	21	16	25	5	6	51
PDL-1 TC%	0.1	0.5	0.1	2.5	0.5	0.2	0	0	2
PDL-1 CPS	50.1	23.5	13.1	23.5	16.5	25.2	5	6	53
PD1 IC%	36	25	29	20	41	31	35	21	34
Death	no	no	no	no	no	no	no	no	no
Recurrence**	Jan 2019 (PUL)	no	no	no	no	no	no	no	Aug 2017 (THO) Feb 2018 (OSS) May 2018 (HEP)

CUP/iCRC = Cancer of unknown Primary, immunophenotypically colorectal carcinoma; MS-stability = microsatellite stability; MSS = microsatellite stable, WT = wild type, Jan = January, Feb = February, Mar = March, Aug = August, Oct = October, Nov = November, THO = thorax, OSS = osseous, HEP = hepatic * details of systemic therapy in Additional file 2: Table S6), ** recurrence after resection of analyzed hepatic metastasis

### Tissue slice culture system

Immediately after surgery, the metastasectomy specimens were transported to the Institute of pathology. Viable tissue (length: 10 mm; diameter: 6 mm) from the invasive margin of the metastasis was sampled using a punch tool (KAI Medical Biopsy Punch, Solingen, Germany) and stored in 4 °C chilled Krebs-Henseleit-Buffer (Sigma-Aldrich/Merck, Darmstadt, Germany). In order to confirm the extraction of adequate tumor tissue, a 1 mm disc was removed with a scalpel from one end of the punch and evaluated in frozen section by a pathologist. Samples without viable tumor were discarded. Punches were then aligned, mounted and immobilized using an agar-ring and cut into thin homogenous slices of 300 μm thickness using a Vibratome VT1200 (Leica Microsystems). They were collected in 4 °C chilled Krebs-Henseleit-Buffer and randomized before distribution to control and therapy groups. The vibration amplitude was adjusted according to the tissue consistency and set between 1 and 2.5 mm. The cutting-velocity was set to 0.4 mm/s. Tissue slices were cultured on special cell-culture inserts (PET membrane with 0.4 μm pore size, Falcon, Corning, USA) to allow preservation of the 3-dimensional structure and assuring the supply with oxygen and cell medium. DMEM (ATCC, Manassas, USA) cell culture medium supplemented with 1% Penicillin/Streptomycin (Sigma-Aldrich/Merck, Darmstadt, Germany; 10000 U Penicillin + 10 mg/ml Streptomycin in 0.9% NaCl) and 10% Fetal Calf Serum (Sigma-Aldrich/Merck, Darmstadt, Germany) was used. For additional oxygen supply, plates were put on an orbital shaker (Thermo Scientific, MaxQ2000 CO_2_ Plus, 55 rpm) during incubation. Incubation was performed at 37 °C under atmospheric oxygen and CO_2_ levels. Medium (with or without systemic agents) was changed after 1 hour and every additional 24 h. After 72 h of incubation, tissue slices were harvested and fixed in 4% buffered formalin for a maximum of 24 h. The time between the end of surgery and the start of cultivation of the tumor tissue slices should be as low as possible and was in our case minimally 2 h and maximally 4 h (median 3 h).

### Treatment regimen

Tissue slices were treated with two concentrations of Oxaliplatin (5 and 20 μM); Cetuximab (20 and 200 nM) and Pembrolizumab (140 and 1400 nM). Concentrations were chosen based on already published cell-culture experiments and recent clinical trials [16–20]. In order to account for tumor-heterogeneity, cultivation was performed in quadruplets (*n* = 4) for each drug and concentration. Twelve tissue slices (*n* = 12) were used for the untreated control group. Due to the small size of the liver metastasis, only triplicates were used in case of patients 9 and 4, respectively.

### Conventional and immunohistochemical staining

Tissue slices were paraffin embedded and processed to 2 μm sections by a microtome for morphological and immunohistochemical evaluation. For morphological analyses, sections were stained with Hematoxylin and eosin (H&E) and with Elastika-van-Gieson (EvG) according to manufacturer specifications (Roth, Karlsruhe, Germany). Proliferation activity was evaluated using the immunohistochemical surrogate marker Ki-67. Apoptotic indices were assessed using cleaved Caspase 3 (Casp 3) immunostaining. In addition, key-proteins of the checkpoint inhibition system PD1 and PD-L1 were stained on whole slides of the routine-diagnostic sections. Furthermore, microsatellite stability was evaluated using immunohistochemical evaluation of MLH1 and MSH2. Prior to immunostaining sections were dewaxed (30 min at 60 °C; 3 × 5 min Xylol) and rehydrated (decreasing alcohol concentration 100 to 50% Ethanol, each 3 min). Staining was performed automatically using the Dako EnVision™ FLEX HRP/DAB; K 8010 Kit (Dako, Agilent, Santa Clara, USA) and the BenchMark ULTRA platform (Ventana Medical Systems, Oro Valley, USA) according to the manufacturer’s specifications. All buffers and chemical agents were included in the kit. While the primary antibodies Ki-67 (Dako Ref.: IR626, mouse), MLH1 (Dako Ref.: IR079, mouse) and MSH2 (Dako Ref.: IR085, mouse) were ready to use, PD1 (Abcam, ab52587, mouse) was diluted 1:100, PD-L1 (Abcam, ab213524, rabbit) was diluted 1:250 and Casp 3 (Cell Signaling, Ref: 05/2017, rabbit) was diluted 3:250. All sections were heated for 35 min in a steam cooker at pH 6 (citrate-buffer; Ki-67, PD1, PD-L1, Casp 3) or pH 9 (EDTA; MSH2, MLH1) for antigen retrieval.

### Analysis of RAS and BRAF- mutation

For DNA extraction, an adequate paraffin block was selected by an experienced pathologist (DW). Up to 10 unstained sections (thickness: 5 μm) of each block were manually macrodissected to enrich tumor cells. Tumor cell content ranged from 50 to 80%, with a median cellularity of 60%. DNA was isolated using RSC DNA FFPE PLUS Custom Kit AX 4920 Promega (Wisconsin, USA) and quantified using Nano Drop (Avantor, Pennsylvania, USA). RAS mutations were analyzed using PCR-based Sanger sequencing. Following primers were used:

*NRAS Gene Exon 2*

- NRAS-F 5′-GATGTGGCTCGCCAATTAAC-3′
- NRAS-R 5′-CCGACAAGTGAGAGACAGGA-3′
- NRAS-RN 5′-GATCAGGTCAGCGGGCTA-3′

*NRAS Gene Exon 3*

- NRAS-F 5′-CCCCTTACCCTCCACACC-3′
- NRAS-R 5′-GAACACAAAGATCATCCTTTCAGA-3′
- NRAS-RN 5′-CCTTTCAGAGAAAATAATGCTCCT-3′

*NRAS Gene Exon 4*

- NRAS-F 5′-TGTTCTGATAATATATTCCCGT-3′
- NRAS-R 5′-GCACTCCAGCTTAGAAGATA-3′
- NRAS-RN 5′-GGATCACATCTCTACCAGAG-3′

*KRAS Gene Exon 2*

- KRAS-F 5′-GGTGAGTTTGTATTAAAAGGTACTGG-3′
- KRAS-FN 5′-TTAACCTTATGTGTGACATGTTCTAA-3′
- KRAS-R 5′-GGTCCTGCACCAGTAATATGC-3′
- KRAS-RN 5′-AAAACAAGATTTACCTCTATTGTTGGA-3′

*KRAS Gene Exon 3*

- KRAS-F 5′-TCCAGACTGTGTTTCTCCCT-3′
- KRAS-R 5′-AACCCACCTATAATGGTGAATATC-3′
- KRAS-RN 5′-TTTATGGCAAATACACAAAGAAAG-3′

*KRAS Gene Exon 4*

- KRAS-F 5′-TTTTTCTTTCCCAGAGAACAAAT-3′
- KRAS-R 5′-AGCATAATTGAGAGAAAAACTGA-3′
- KRAS-RN 5′-ACATAACAGTTATGATTTTGCAG-3′

*BRAF Gene Exon 15*

- BRAF-F 5′- ATCTCTTACCTAAACTCTTCATAATGC -3′
- BRAF-R 5′- GGCCAAAAATTTAATCAGTGGA-3′

The sequencing results were interpreted using Genome Lab GeXP Genetic Analysis System (Beckman Coulter, California, USA).

### Analysis of tissue slice culture

**Fig. 1:**
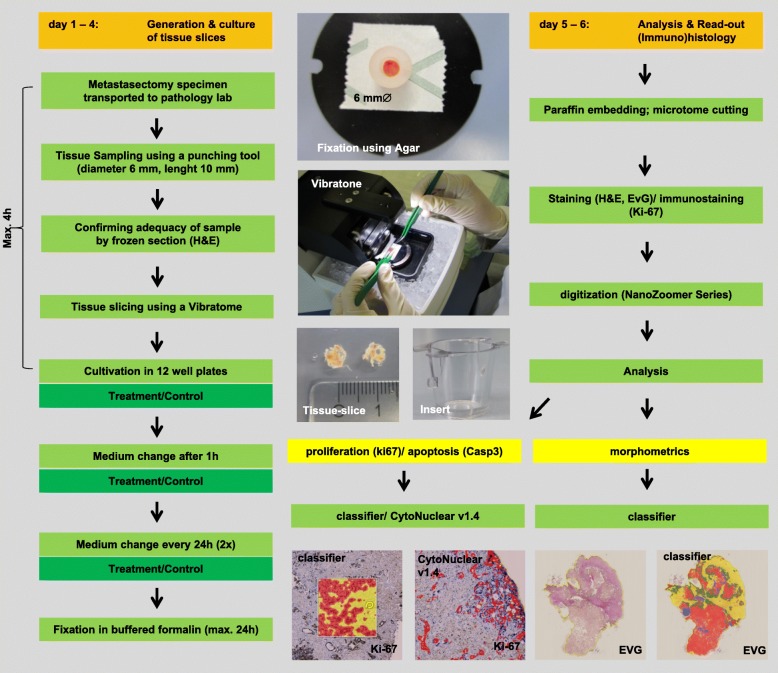
Experimental setup of the tissue slice culture system. Susceptibility to systemic drugs is assessed within 6 days

All sections were digitalized using the NanoZoomer-Series Digital Slide Scanner (40×, Hamamatsu Photonics, Hamamatsu, Japan). Firstly, H&E stained untreated tissue slices (controls) were visually compared with their representative paraffin-embedded sections used in routine-diagnostic by a pathologist. Overall morphological appearance, architecture, growth-patterns, grading of differentiation and nuclear characteristics of the tumor were assessed. Secondly, untreated (control) and treated (Oxaliplatin, Cetuximab, Pembrolizumab) tissue slices were compared using an automated analysis-readout based on the Halo platform from Indica Labs (Corrales, NM, USA). For immunohistochemical analysis of Ki-67 the module CytoNuclear v1.4 was applied. In a training phase, five representative sections were used to define staining parameters (e.g. minimum nuclear optical density, minimum staining optical density, nuclear and cellular size and roundness) for an optimal distinction between Ki-67 positive and negative tumor cells. A tissue classifier was then trained separately for each section to select epithelial tumor cells. Stroma, blood vessels and areas of necrosis were excluded from analysis. The percentage of Ki-67-positive tumor cells in relation to the total number of tumor cells was calculated and used as a surrogate marker for the proliferation activity. All automated results were visually validated for accuracy. For morphometrical analysis, the EvG stain was used. For each tumor tissue slice a Halo tissue-classifier was trained to recognize the stromal, tumor and necrosis compartment. The area of each compartment was calculated and normalized to the total analyzed area. The automated results were visually validated. For immunohistochemical analysis of Casp 3 digitized slides were visually assessed semiquantitatively by two experienced pathologist (SZM, WR). The apoptotic state is expressed as the tumor-apoptotic fraction defined as the number of Casp 3 positive tumor cells divided by the total number of tumor cells. Importantly, dependent on the cells stage of apoptosis, Casp 3 stain can be nuclear or cytoplasmatic [21]. Stain of non-epithelial cells, necrosis or cell debris was excluded (see Additional file 1). Tissue slices that showed no tumor were excluded from analysis (7 slices of 312). Figure 1 depicts the experimental set up of the tissue slice culture system.

### Proteins of the checkpoint inhibition system

Immunohistochemical PD-L1 and PD1 positivity was analyzed based on the urothelial carcinoma PD-L1 interpretation manual of Agilent [22]. In short, staining of the cell-membrane was classified as positive. Whole slides of the routine-diagnostic sections were assessed visually and positive immune cells and tumor cells were determined in areas comprising approximately 100,000 tumor cells. Necrosis and cell-debris were excluded. Combined positivity score (CPS), tumor cell score (TC%) and immune cell score (IC%) were defined as follows and are depicted in Table 1:

$$ CPS=\frac{PDL-1\  positive\ tumor\ cells+ PDL-1\  positive\ immune\ cells}{total\ count\ of\ tumor\ cells}\ x\ 100 $$

$$ TC\%=\frac{PDL-1\  positive\ tumor\ cells}{total\ count\ of\ tumor\ cells}\ x\ 100 $$

$$ IC\%=\frac{PDL-1\  positive\ immune\ cells}{total\ count\ of\ tumor\ cells}\ x\ 100 $$

### Statistical analysis

Analysis of morphometry, Ki-67-proliferation and Casp 3 apoptotic state was performed across all patients and for each patient individually. For the pooled analysis of the Ki-67-proliferation and Casp 3 apoptotic fraction and morphometry, the mean values of each patient were used and depicted in Box-Jitter plots. Statistical significant differences between the control and treatment groups were calculated using the nonparametric Mann–Whitney U test. *P*-values ≤0.05 were defined as significant. Additionally, the analysis was performed for each patient individually. To calculate differences between the control and treatment groups in each patient, the nonparametric Mann–Whitney U test was performed. Wilcoxon-signed rank test for paired samples can only be used, if the number of tissue slices each group are equal in number. Since this requirement is not met in our study (treatment group *n* = 4, control group *n* = 12), the *p*-value of the Mann-Whitney U test gives the best approximation of statistical relevant group differences. For Ki-67-analysis, a representative 1 mm^2^ area of the routine-diagnostic section was included in the analysis. For the morphometrical analysis, the medians of the area of necrosis, tumor and stroma for each patient were depicted in stacked plots and normalized to the total area. For statistical analysis, the software Past Version 3.16 [23] was used.

## Results

### Tissue slice culture

**Fig. 2:**
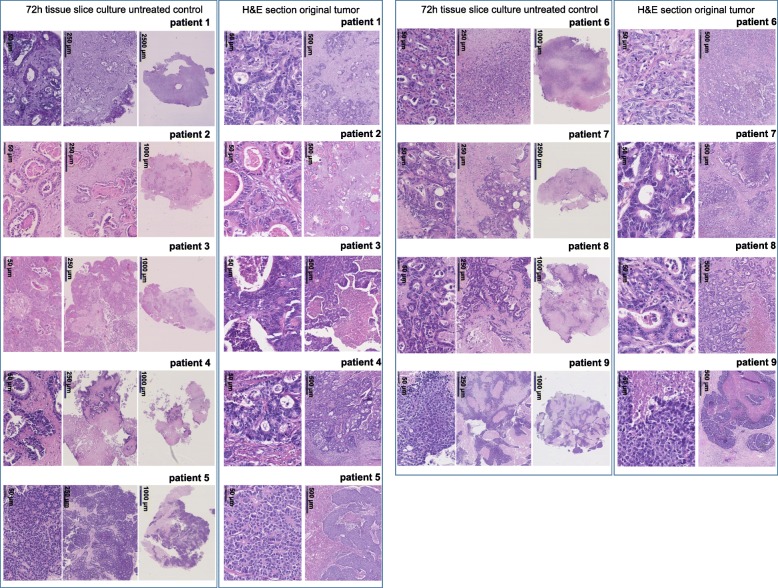
Depicted are H&E stained sections of the original tumor tissue and representative untreated tissue slices (control) that were cultured for 72 h. The upper part shows the original tumor (routine-diagnostics) in high magnification. Tissue slices are depicted in the lower part in high magnification

**Fig. 3:**
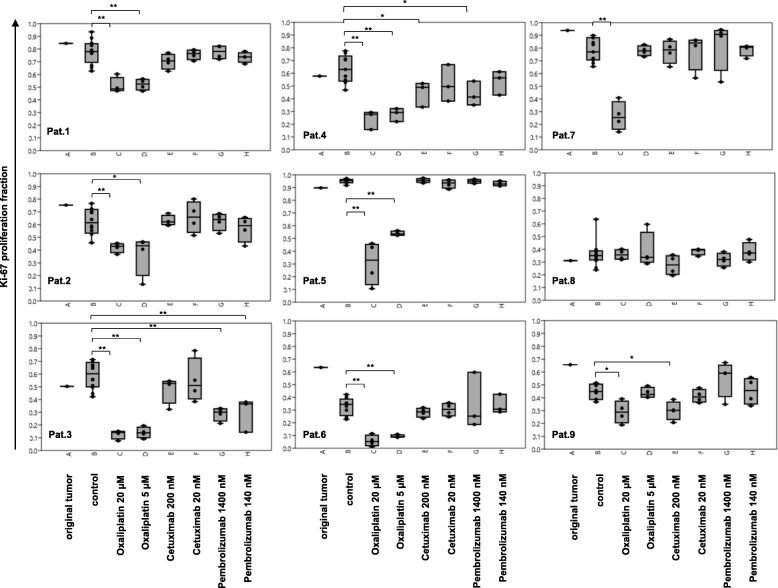
Tumor- proliferative activity (Ki-67) of treated (Cetuximab, Pembrolizumab and Oxaliplatin) and untreated (control) tissue slices. Additionally, one 1 mm^2^ representative section of the original tumor tissue was included in the analysis (routine-diagnostic). The percentage of Ki-67 positive tumor cells is depicted in Box-Jitter plots. Statistical differences were calculated using the Mann-Whitney U test and are marked (* *p* value ≤0.05; ** *p* value ≤0.01). **a**- original tumor; **b**- control; **c**- Oxaliplatin 20 μM; **d**- Oxaliplatin 5 μM; **e**- Cetuximab 200 nM; **f**- Cetuximab 20 nM; **g**- Pembrolizumab 1400 nM; **h**- Pembrolizumab 140 nM

The tumor tissue slice culture technique was adjusted for liver metastases of colorectal cancer patients. Tumor tissue from nine metastases was cultured for 72 h and morphologically compared to representative routine-diagnostic H&E sections from the original tissue (see Fig. 2). There was a high morphological similarity between the ex vivo and in vivo tumor, as evidenced by comparable tumor growth-patterns, architecture, grading of differentiation and tumor cell cytology. The tumor of tissue slices exhibited only minimal heterogeneous nuclear changes like karyorrhexis, karyolysis or pyknosis in some tumor glands. The immunohistologically assessed proliferation activity (Ki-67) showed a moderate reduction in proliferation for tumors of patients 1, 2, 6, 7 and 9 and similar proliferation for tumors of patients 3 to 5, when comparing the untreated tissue slices with a representative 1 mm^2^ area of the original tumor (see Fig. 3).

### Readout of proliferation index and apoptotic index

**Fig. 4:**
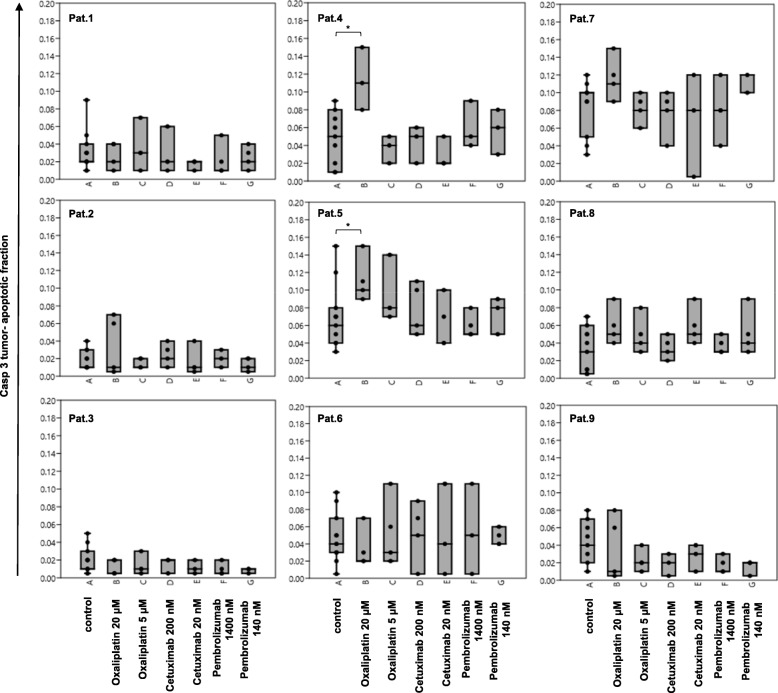
Tumor- apoptotic- fraction (Casp3) of treated (Cetuximab, Pembrolizumab and Oxaliplatin) and untreated (control) tissue slices. The percentage of Casp3 positive tumor cells is depicted in Box-Jitter plots. Statistical differences were calculated using the Mann-Whitney U test and are marked (* p value ≤0.05). **a**- control; **b**- Oxaliplatin 20 μM; **c**- Oxaliplatin 5 μM; **d**- Cetuximab 200 nM; **e**- Cetuximab 20 nM; **f**- Pembrolizumab 1400 nM; **g**- Pembrolizumab 140 nM

The tumor tissue slice culture technique was used to measure drug responses of metastatic colorectal cancer tissue. Tumor tissue was treated with Oxaliplatin (5 and 20 μM), Pembrolizumab (140 and 1400 nM) and Cetuximab (20 and 200 nM) for 72 h and compared to untreated controls. To measure susceptibility to those drugs an automated analysis of the proliferation index using Ki-67 immunostain was performed for each patient individually (Fig. 3, Additional file 2: Table S1 and Additional file 3). Additionally semiquantitative analysis of the apoptotic index was carried out using Casp 3 immunostain (Fig. 4, Additional file 2: Table S2 and Additional file 3).

Proliferation activity of the untreated tissue slices were heterogeneous and varied between 95% in case 5 and 34% in case 6 (median value of 60 ± 19%). Regarding the original tumors proliferative activity ranged from 94% in case 7 to 31% in case 8 (median value of 65 ± 19%). Tumors of patients 1 to 6 showed a reduction of the Ki-67- positive tumor fraction when treated with 5 μM and 20 μM Oxaliplatin. Tumors of patients 7 and 9 showed a reduction only when treated with 20 μM Oxaliplatin. A dosage dependent decrease of proliferation was visible for tumor of patient 5 (95% control, 53% 5 μM and 33% 20 μM Oxaliplatin). The absolute difference of the medians between the untreated (control) and treated (20 μM Oxaliplatin) group was ranging from 62% (patient 5) to 16% (cases 2 and 9) or 0% (case 8). Only tumors of patients 3, 4 and 9 showed a reduction in proliferation, when treated with Pembrolizumab or Cetuximab. Tissue of patient 3 showed a median drop of 23 and 30% when treated with Pembrolizumab (140 and 1400 nM respectively), which was smaller compared to the Oxaliplatin treatment (46% for both concentrations). Tumor of patient 4 showed a decrease in Ki-67 positivity when treated with 200 nM Cetuximab (14%) or 1400 nM Pembrolizumab (22%). Again, this reduction was lower than in the Oxaliplatin-treated group (drop of 35% for both concentrations). Tumor of patient 9 showed a median reduction of the proliferation index of 15%, when treated with 200 nM Cetuximab, which was as high, as in the Oxaliplatin-treated group. Tumor of patient 8 showed no differences in proliferation between control and treatment groups. The tumor-apoptotic fraction of the untreated tissue slices were also heterogeneous and varied between 1% (case 2) and 9.5% (case 7). Tumors of patients 4 to 5 showed an increase of the Casp 3- positive tumor fraction when treated with 20 μM Oxaliplatin. All other treatment groups showed no statistically relevant differences compared to the control group.

**Fig. 5:**
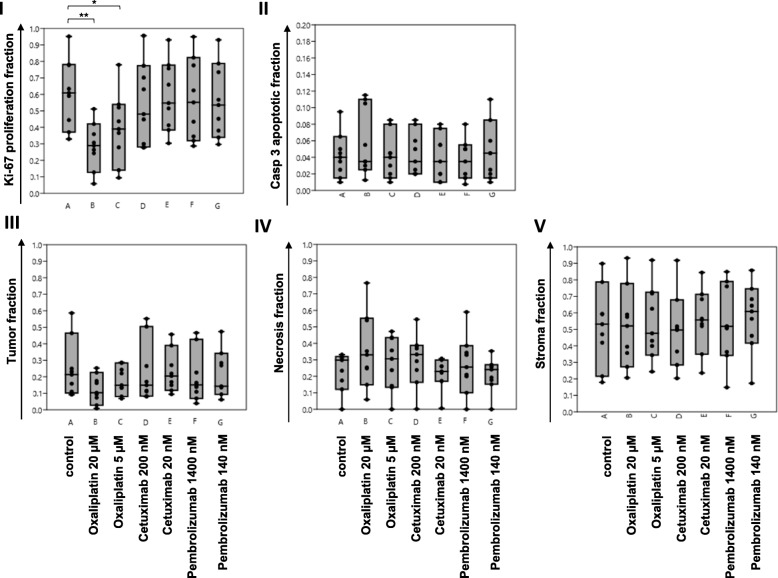
Depicted are tumor-proliferative fractions (I), tumor-apoptotic fractions (II), tumor (III), necrosis (IV) and stroma (V) fractions of Ki-67, Casp3 and morphometric analysis across all nine patients in Box-Jitter plots. The mean-values of each patient are depicted as a black dot. Statistical differences were calculated using the Mann-Whitney U test and are marked (* p value ≤0.05; ** p value ≤0.01). **a**- control; **b**- Oxaliplatin 20 μM; **c**- Oxaliplatin 5 μM; **d**- Cetuximab 200 nM; **e**- Cetuximab 20 nM; **f**- Pembrolizumab 1400 nM; **g**- Pembrolizumab 140 nM

Pooled analysis of the Ki-67 proliferation fraction across all nine cases confirmed a statistical significant and dosage dependent reduction when treated with Oxaliplatin. There were no significant differences in proliferation after treatment with Pembrolizumab or Cetuximab. Pooled analysis of the Casp 3 tumor-apoptotic fraction across all nine cases revealed no statistical significant differences between control and treatment groups (see Fig. 5).

### Automated readout of morphometrical analysis

**Fig. 6:**
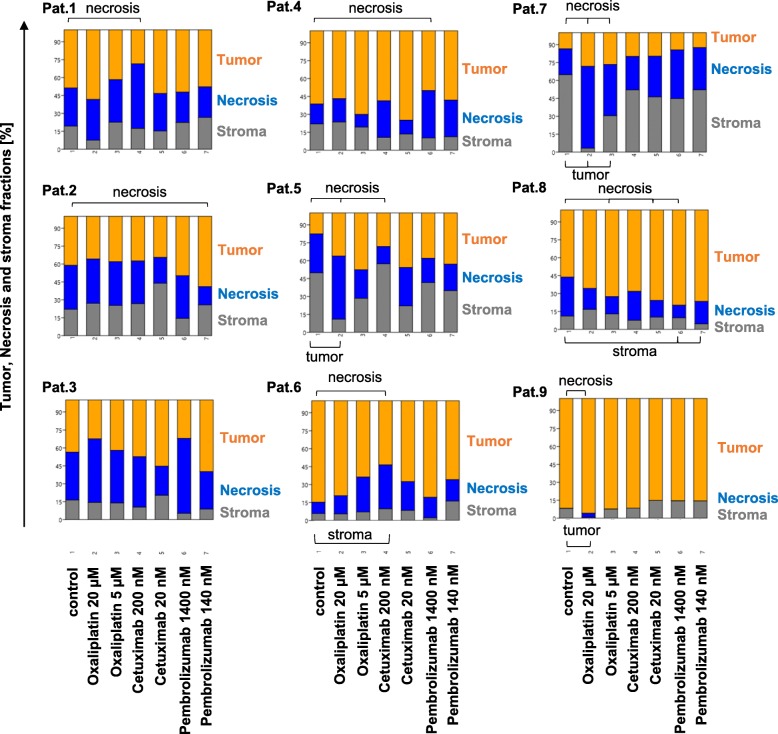
Morphometrical analysis of the treated (Cetuximab, Pembrolizumab and Oxaliplatin) and untreated (control) tissue slices. Stacked plots show the medians of the areas of necrosis (blue), stroma (orange) and tumor (grey) normalized to the total area analyzed. Statistical differences between the groups were calculated using the Mann-Whitney U test and marked with a parenthesis and a label (*p* ≤ 0.05). 1- control; 2- Oxaliplatin 20 μM; 3- Oxaliplatin 5 μM; 4- Cetuximab 200 nM; 5- Cetuximab 20 nM; 6- Pembrolizumab 1400 nM; 7- Pembrolizumab 140 nM

In addition to the evaluation of proliferative changes after drug treatment, also morphometric changes were assessed. To measure variations of the area of necrosis, stroma and tumor, treated and untreated tissue slices were stained with EvG and quantified using the Halo-platform. In contrast to H&E, EvG showed a superior contrast between necrosis and stroma in direct comparison and led to a more accurate distinction using the Halo classifier (data not shown). Findings of the analysis are depicted in Fig. 6 and Additional file 2: Table S3). The morphometric analysis of the untreated tissue slices showed substantial differences in the distribution of necrosis, tumor and stroma for all 9 cases. While tumor of patient 3 showed the highest amount of necrosis (median 37%), tumor of patient 9 showed no necrosis at all. An increase in necrosis accompanied by a reduction of the tumor area was visible for cases 5 and 9 when treated with 20 μM Oxaliplatin and for case 7 when treated with 5 and 20 μM Oxaliplatin. Tumors of patients 1 and 6 showed an increase in necrosis after treatment with 200 nM Cetuximab, in case of patient 6 accompanied by a reduction of the stromal compartment. Tumor of patient 4 showed an increase in necrosis when treated with 1400 nM Pembrolizumab. Tumor of patient 3 showed no differences among the groups. Tumors of patients 2 (Pembrolizumab), 5 (Cetuximab) and 8 (Oxaliplatin, Cetuximab and Pembrolizumab) showed a reduction of areas of necrosis, in case of patient 8 accompanied by an increase of the stromal compartment (Pembrolizumab). Pooled morphometric analysis across all nine cases showed no statistically significant differences in necrosis, stroma or tumor-area between control and treatment groups (see Fig. 5).

### Associations between drug response and molecular tumor characteristics

In order to determine associations of therapy response with molecular tumor characteristics, the RAS mutation status as well as the immunohistochemical evaluation of microsatellite stability and checkpoint protein expression was assessed.

Visual semiquantitative analysis in whole slides of the original tumor sections showed moderate to high infiltrates of PD1 positive tumor-associated immune cells for all cases particularly at the invasive margin. The PD1 immune cell score (IC%) ranged from 20 to 36 (see Table 1 and Additional file 2: Table S4). PD-L1 analysis showed only few positive tumor cells and moderate to high infiltrates of PD-L1 positive tumor-associated immune cells, particularly at the invasive margin. Only tumors of patients 4 and 9 showed a tumor cell score (TC%) above 1. CPS scores were above 10 for the cases 1–6 and 9 and below 10 for the cases 7 and 8 (see Table 1 and Additional file 2: Table S5). Of all cases, only tumor tissue of patients 3 and 4 showed a reduction of proliferation when treated with Pembrolizumab.

All cases showed immunohistochemical expression of MLH1 and MSH2 and therefore no sign of microsatellite instability.

PCR-based Sanger sequencing showed KRAS mutations in metastatic tumor tissue of patients 2 (G12D), 3 (G12A) and 4 (G13D) and a NRAS mutation for patient 8 (G13R “c37G > C). Of the five cases harboring no RAS-mutations, only tumor tissue of patient 9 showed a reduction of proliferative activity after treatment with Cetuximab. Additionally, tumor tissue of patient 4, harboring a G13D KRAS mutation, showed a response after cultivation with Cetuximab.

## Discussion

In this study, we present an experimental ex vivo test system based on the tissue slice culture approach to estimate the susceptibility of colorectal liver metastases to different drugs. The tissue slice culture approach allows for cultivating tumor tissue, while preserving the tumor morphology and microenvironment. Keeping stromal-tumor interactions intact is fundamental, because they are known to affect progression, proliferation and sensitivity to drugs [24, 25]. Since every tumor entity carries its own stromal-tumor microenvironment, optimal tissue slice culture protocols must be identified for each type separately. So far, only the tissue of the primaries of breast, prostate, lung, colorectal, gastroesophageal and head- and neck carcinomas have been successfully cultured for several days [16, 26–32]. Hepatic colorectal metastasectomy specimens are a major challenge for the tissue slice culture technique, since they show extensive regressive changes. This study is the first to successfully cultivate colorectal liver metastatic tissue for up to 72 h, keeping stromal-tumor interactions intact and preserving the in vivo tumor morphology (for details of cultivation protocol see Methods and Additional file 4). Additionally, analysis of the proliferation activity showed only moderate if any differences between the in vivo (original tumor) and ex vivo (tissue slice) tumor. Although the technical, organizational and personnel requirements of the method will probably surpass the capabilities of some pathological institutes, implementation at larger and specialized university centers is easily possible.

The construction of a predictive ex vivo test system requires an objective and easy to use read out strategy. In this study, we used an automated analysis tool based on digital image analysis. Changes in proliferative activity of the tumor cells were measured using Ki-67-immunostaining as a surrogate marker. Median values were 60 ± 19% for untreated tissue slices and 65 ± 19% for original tumors, as was reported before [33, 34]. Changes in the tumor-stroma-necrosis make-up of the tissue slices were measured using EvG-stains. Halo classifiers had to be trained for each section and the analysis visually validated by a pathologist (SZM) to be repeated if necessary. This automated procedure took about 5 min per section and is approximately as time-consuming as a purely visually semi-quantitative method. The essential advantages, however, are a reliable absolute quantification of the entire section in a short period of time and a high objectivity and reproducibility of the procedure.

Tumor tissue slices were treated with Oxaliplatin, Pembrolizumab and Cetuximab, all of which play an important role in the treatment of metastatic colorectal cancer. Oxaliplatin is a cytostatic drug that interlinks DNA-strands inhibiting replication [35] and represents in combination with fluorouracil and leucovorin the first-line standard therapy for metastatic colorectal carcinoma [36]. As a single agent, it demonstrates modest activity with response rates of 10 to 25% [37–40], which was confirmed in our study. All but one patients showed a dose-dependent reduction of the proliferative activity that was additionally confirmed in a pooled analysis across all nine patients. The morphometric analysis showed increases in areas of necrosis for cases 5, 7 and 9, which was partially accompanied by a decrease in the tumor area.

Cetuximab is a monoclonal antibody against the epidermal growth factor receptor EGFR and is often added to first line therapy to improve outcome [4, 10, 41]. It exerts its biological anti-tumor effects in two ways. On the one hand, the EGFR signaling pathway on tumor cells is specifically blocked, leading to cell cycle arrest, reduction of tumor-cell proliferation and increase of apoptosis [42]. Therefore, mutations in the RAS gene are predictive for treatment failure. Of the five cases harboring no RAS-mutations in our study, only tumor of patient 9 seemed to be sensitive towards Cetuximab. Possible reasons for treatment resistance in those other patients are mutations of genes downstream the EGFR/RAS signaling cascade that are, although recommended, not regularly evaluated before systemic therapy in clinical practice [43]. This supports that molecular profiling alone cannot always accurately predict response to therapy in a clinical setting and underlines the need of additional predictive test systems. The tumor of patient 4 is the only one that harbored a RAS mutation while showing intermediate reduction of the proliferative activity after treatment with Cetuximab. However, this specific G13D mutation in the KRAS-gene was shown to be sensitive to Cetuximab treatment in a retrospective trial [44, 45] and in in-vitro cell-culture [46]. On the other hand, being an IgG1 antibody, Cetuximab can crosslink with and activate immune cells via its constant (Fc) region to induce antibody-dependent cellular cytotoxicity (ADCC) [47, 48]. While tumor tissue slices are thought to preserve the immune compartment of local native immune cells during the cultivation process, the suitability of this technique to study ADCC effects has not been investigated so far and exceeded the scope of this study.

Pembrolizumab is a monoclonal antibody to programmed cell death 1 protein and FDA approved as second-line therapy for unresectable metastatic colorectal cancer that has high microsatellite instability or deficient mismatch repair [49–53]. Recent data supports that tumors harboring other mutations of DNA proofreading enzymes (e.g. POLE) also upregulate expression of immune checkpoints and are eligible to checkpoint inhibition, while showing an MSS Immunophenotype [52, 53]. Those mutations might explain the reduction of proliferation of tumors of patients 3 and 4 when treated with Pembrolizumab. However, only patient 4 showed a high PD-L1 TC% score above 1. Whether PD-L1 immunostaining is indeed predictive for response to Pembrolizumab therapy in colorectal carcinoma is still unknown and needs to be evaluated in adequate prospective trials [51].

The apoptotic state of the tumor tissue was revealed using Casp 3 immunostain, which is a well-known and highly sensitive method to visualize different steps of the apoptotic process [21]. A significant increase in the number of apoptosis of tumor cells was only detected for tumor tissue of patients 4 and 5, when treated with the cytotoxic drug Oxaliplatin, but was not confirmed in pooled analysis. This finding is supported by recent data of Buzzelli et al. who established colorectal cancer liver metastases organoids and observed that while organoids showed growth delay in response to Oxaliplatin treatment, they did not undergo significant cell death [54]. Both, this finding and our data suggest that Oxaliplatin limits tumor growth through reduction of proliferation and not by inducing apoptosis, which is consistent with its known function to inhibit DNA synthesis. Treatment of tumor tissue with Cetuximab and Pembrolizumab did not reveal a significant increase in the tumor-apoptotic fraction in this study. While there is no data available for colorectal cancer tissue, Gerlach et al. did also not detect significant changes in the number of Casp 3 positive cells after treatment of Head and Neck carcinoma tissue slices with Cetuximab [32].

In summary, the findings of this study suggest a direct correlation between the reduction of the proliferative tumor fraction and the level of drug sensitivity. Therefore, the tumor tissue slice culture approach seems feasible for measuring drug-responses and should be evaluated in further co-clinical trials. Here, a tumor tissue sample is processed in tissue slice culture before systemic therapy, allowing for subsequent direct comparison of ex vivo and in- vivo determined response rates.

There are several limitations to this study. Firstly, Oxaliplatin was used as a single agent rather than in combination with 5-FU. Also, only nine patients have been enrolled and investigated that were heterogeneous in their clinical characteristics regarding presurgical therapy, stage and localization of the primary tumor. In addition, there was no data available about the in vivo response rates to systemic therapy, as would be necessary for a co-clinical trial design. Furthermore, we have only performed an automated analysis of changes in morphometrics and proliferative activity via Ki-67. Since Casp 3 immunostain localization can be either cytoplasmatic or nuclear, depending on the apoptotic stage of the individual tumor cell, automated analysis was not possible, because all available digital modules rely on a sole nuclear or cytoplasmatic/ membranous localization of the immune stain. Therefore, evaluation was performed semiquantitatively by two experienced pathologists.

## Conclusion

We showed that the tissue slice culture technology is feasible for conserving tumor-stroma morphology of hepatic metastases of colorectal cancer. Easy to use automated analysis tools objectively measure absolute changes of proliferation and the distribution among necrosis-, tumor- and stroma- compartments after treatment with systemic drugs. Therefore, this study indicates a potential value of this technique as a patient-specific test-system of targeted therapy in the context of metastatic colorectal carcinoma. Future co-clinical trials will test this hypothesis and define adequate cut-off values of the readout data.

## Supplementary information

**Additional file 1: Figure S1.** Depicted are examples of selective nuclear and cytoplasmatic location of Casp 3 immunostain of tumor cells (upper row), depending on the individual stage of apoptosis. The tumor apoptotic fraction is defined as Casp 3 positive tumor cells divided by the total number of tumor cells. Stain of non-epithelial cells or unspecific stain of cell debris and necrosis (middle row) were ignored. The lower left picture shows a section detail with three tumor cells positively stained for Casp 3 (black arrows) and unspecific stain (red arrows).

**Additional file 2:** **Supplementary Tables.** Description of data: Tables with raw data of immunohistochemical and morphometrical analysis as well as evaluation of PD1 and PD-L1 immunostain and details of systemic therapy.

**Additional file 3: Figure S2.**. Depicted are H&E, EvG, Ki-67 and Casp 3 stained sections of representative treated (Cetuximab, Pembrolizumab and Oxaliplatin) and untreated (control) tissue slices of patient 5. The upper row depicts H&E stained sections, little boxes show a higher magnification to show nuclear detail. The middle row shows EvG-stained sections and Ki-67 immunostain. The lower row shows Casp 3 Immunostain, little boxes show a higher magnification to show nuclear detail.

**Additional file 4:** Adaptations to Tumor Tissue Slice Culture for Hepatic Colorectal Metastases. More detailed information of the protocol of tumor tissue slice culture is provided.

## Data Availability

All data generated or analyzed during this study are included in this published article and its supplementary information files, with the exception of data that would compromise the individual privacy of the patients.

## Abbreviations

ADCC: antibody-dependent cellular cytotoxicity
Casp 3: cleaved caspase-3
CT: Computed Tomography
CUP: Cancer of unknown Primary
DW: Daniel C. Wagner
EvG: Elastica van Gieson
F: Fluorouracil
FOL: Folinic Acid
H&E: Hematoxylin and Eosin
MSI: Microsatellite instable
MSS: Microsatellite stable
OX: Oxaliplatin
PET-CT: Positron emission tomography–computed tomography
RECIST: Response Evaluation Criteria In Solid Tumors
SUV: Standardized Uptake Value
SZM: Steve Z. Martin
WR: Wilfried Roth
WT: Wild type
