# Supplementary material for: Ex vivo tissue slice culture system to measure drug-response rates of hepatic metastatic colorectal cancer
Source: BMC Cancer. 2019 Nov 1;19:1030. doi: 10.1186/s12885-019-6270-4 (PMC6824140; doi:10.1186/s12885-019-6270-4)
Supplement: Supplementary file 1 — Additional file 1: Figure S1. Depicted are examples of selective nuclear and cytoplasmatic location of Casp 3 immunostain of tumor cells (upper row), depending on the individual stage of apoptosis. The tumor apoptotic fraction is defined as Casp 3 positive tumor cells divided by the total number of tumor cells. Stain of non-epithelial cells or unspecific stain of cell debris and necrosis (middle row) were ignored. The lower left picture shows a section detail with three tumor cells positively stained for Casp 3 (black arrows) and unspecific stain (red arrows). [file 12885_2019_6270_MOESM1_ESM.pptx]

## Slide 1
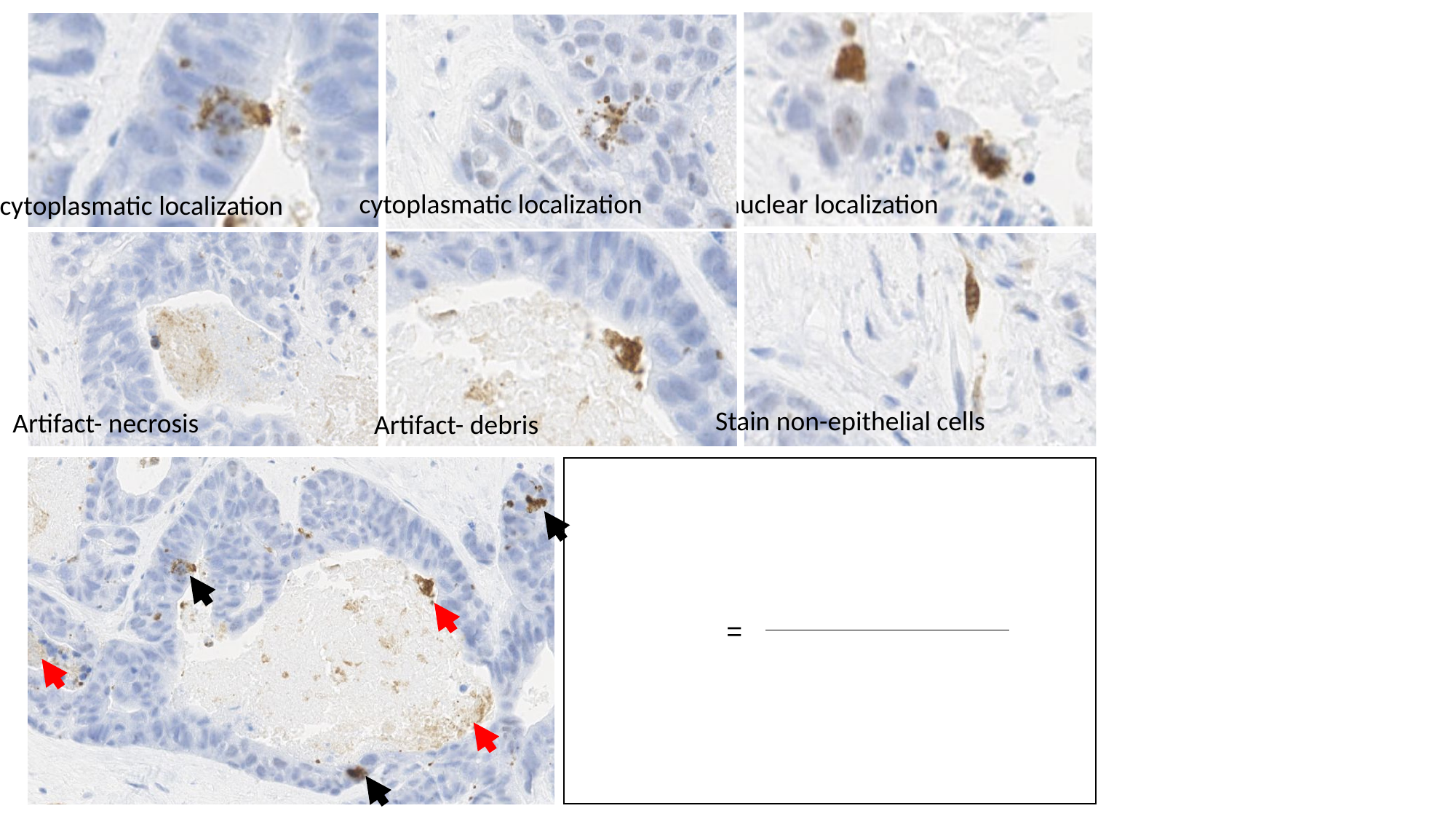

cytoplasmatic localization
nuclear localization
cytoplasmatic localization
Stain non-epithelial cells
Artifact- necrosis
Artifact- debris
=
