# Supplementary material for: Ex vivo tissue slice culture system to measure drug-response rates of hepatic metastatic colorectal cancer
Source: BMC Cancer. 2019 Nov 1;19:1030. doi: 10.1186/s12885-019-6270-4 (PMC6824140; doi:10.1186/s12885-019-6270-4)
Supplement: Supplementary file 2 — Additional file 2: Supplementary Tables. Description of data: Tables with raw data of immunohistochemical and morphometrical analysis as well as evaluation of PD1 and PD-L1 immunostain and details of systemic therapy. [file 12885_2019_6270_MOESM2_ESM.docx]

Supplementary Table 1: Percentages of Ki-67 positive tumor cells for untreated (control) and treated (Oxaliplatin, Cetuximab, Pembrolizumab) tissue slices. In the upper part of the table the following values are depicted: median [minimum; maximum]; standard deviation. The lower part of the table shows p-values of the Mann-Whitney U Test.

| patient | control | Oxaliplatin 5 µM | Oxaliplatin 20 µM | Cetuximab 20 nM | Cetuximab 200 nM | Pembrolizumab 140 nM | Pembrolizumab 1400 nM |
| --- | --- | --- | --- | --- | --- | --- | --- |
| 1 | 78 [63;94]; 9 | 52 [47,57]; 4 | 49 [47, 60]; 6 | 76 [71,80]; 4 | 71 [63,77]; 6 | 74 [68,78]; 4 | 78 [72,82]; 5 |
| 2 | 59 [46;77]; 11 | 43 [13;47]; 16 | 43 [37;45]; 4 | 66 [51;80]; 12 | 62 [59;69]; 4 | 59 [43;66]; 10 | 64 [53;69]; 7 |
| 3 | 60 [42;71]; 11 | 14 [9;19]; 4 | 14 [8;15]; 3 | 51 [38;78]; 17 | 53 [32;55]; 11 | 37 [14;38]; 13 | 30 [21;33]; 5 |
| 4 | 63 [47;78]; 11 | 29 [22;32]; 5 | 28 [16;29]; 7 | 50 [38;67]; 14 | 49 [33;52]; 10 | 56 [43;61]; 9 | 41 [35;54]; 10 |
| 5 | 95 [92;97]; 2 | 53 [52;56]; 2 | 33 [11;46]; 17 | 94 [89;96]; 3 | 96 [94;98]; 2 | 93 [91;95]; 2 | 95 [93;97]; 1 |
| 6 | 34 [23;42]; 7 | 9 [9;11]; 1 | 5 [1;11]; 4 | 31 [25;36]; 5 | 29 [23;32]; 4 | 31 [29;42]; 8 | 25 [19;60]; 22 |
| 7 | 77 [66;90]; 9 | 78 [73;83]; 4 | 25 [14;41]; 11 | 84 [57;86]; 14 | 79 [65;87]; 9 | 81 [72;81]; 5 | 91 [53;94]; 19 |
| 8 | 36 [25;64]; 10 | 34 [29;60]; 14 | 36 [32;40]; 4 | 39 [35;40]; 3 | 28 [19;36]; 8 | 37 [30;48]; 7 | 32 [26;38]; 5 |
| 9 | 45 [37;51]; 6 | 42 [40;49]; 4 | 29 [19;39]; 9 | 41 [36;48]; 5 | 30 [21;39]; 7 | 46 [34;56]; 10 | 59 [35;67]; 14 |
|  |  |  |  |  |  |  |  |
| patient |  | Oxaliplatin 5 µM | Oxaliplatin 20 µM | Cetuximab 20 nM | Cetuximab 200 nM | Pembrolizumab 140 nM | Pembrolizumab 1400 nM |
| 1 | control vs.: | p = 0.0044 | p = 0.0044 | p = 0.6713 | p = 0.1016 | p = 0.3027 | p = 0.8557 |
| 2 |  | p = 0.0223 | p = 0.0050 | p = 0.4727 | p = 0.7441 | p = 0.5569 | p = 0.9480 |
| 3 |  | p = 0.0044 | p = 0.0044 | p = 0.5048 | p = 0.1631 | p = 0.0115 | p = 0.0044 |
| 4 |  | p = 0.0162 | p = 0.0162 | p = 0.1956 | p = 0.0420 | p = 0.2673 | p = 0.0420 |
| 5 |  | p = 0.0044 | p = 0.0044 | p = 0.2493 | p = 0.8557 | p = 0.0787 | p = 0.7618 |
| 6 |  | p = 0.0044 | p = 0.0044 | p = 0.6713 | p = 0.1296 | p = 0.9425 | p = 0.7182 |
| 7 |  | p = 0.9999 | p = 0.0050 | p = 0.9480 | p = 0.7441 | p = 0.9480 | p = 0.2149 |
| 8 |  | p = 0.7441 | p = 0.8447 | p = 0.1704 | p = 0.1027 | p = 0.6477 | p = 0.3275 |
| 9 |  | p = 0.9323 | p = 0.0338 | p = 0.2696 | p = 0.0219 | p = 0.6711 | p = 0.2027 |

Supplementary Table 2: Percentages of cleaved Caspase 3 positive tumor cells for untreated (control) and treated (Oxaliplatin, Cetuximab, Pembrolizumab) tissue slices. In the upper part of the table the following values are depicted: median [minimum; maximum]; standard deviation. The lower part of the table shows p-values of the Mann-Whitney U Test.

| patient | control | Oxaliplatin 5 µM | Oxaliplatin 20 µM | Cetuximab 20 nM | Cetuximab 200 nM | Pembrolizumab 140 nM | Pembrolizumab 1400 nM |
| --- | --- | --- | --- | --- | --- | --- | --- |
| 1 | 2.5 [1,9]; 2 | 3 [1,4]; 2 | 3 [1,7]; 3 | 2 [1,6]; 2 | 2 [1,2]; 1 | 1.5 [1,5]; 2 | 2.5 [1,4]; 1 |
| 2 | 1 [1,4]; 1 | 3.5 [0.5,7]; 3 | 1.5 [1,2]; 1 | 2.5 [1,4]; 1 | 1 [0.5, 4]; 2 | 2 [1,3]; 1 | 1.5 [0.5,2];1 |
| 3 | 1.5 [0.5,5]; 1 | 1.25 [0.5,2]; 1 | 1 [0.5,3]; 1 | 2 [0.5,2]; 1 | 1 [0.5,2]; 1 | 0.75 [0.5,2]; 1 | 1 [0.5,1]; 0.3 |
| 4 | 5 [1,9]; 3 | 11 [8,15]; 4 | 4 [2,5]; 2 | 5 [2,6]; 2 | 2 [2,5]; 2 | 5 [4,9]; 3 | 6 [3,8]; 3 |
| 5 | 6.5 [3,15]; 4 | 10.5 [9,15]; 3 | 8 [7,14]; 3 | 8 [5,11]; 3 | 5.5 [4,10]; 3 | 5.5 [5,8]; 1 | 8.5 [5,9]; 2 |
| 6 | 4 [0.5,10]; 3 | 2.5 [2,7]; 2 | 4.5 [2,11] 4 | 6 [0.5,9]; 4 | 7.5 [0.5,11]; 5 | 5 [0.5,11]; 5 | 4.5 [4,6]; 1 |
| 7 | 9.5 [3,12]; 3 | 11.5 [9,15]; 3 | 8.5 [6,10]; 2 | 8.5 [4,10]; 3 | 8 [0.5,12]; 5 | 8 [4,12]; 3 | 11 [10,12]; 1 |
| 8 | 3.5 [0.5,7];3 | 5.5 [4,9]; 2 | 4.5 [3,8]; 2 | 3.5 [2,5]; 1 | 5.5 [4,9]; 2 | 3.5 [3,5]; 1 | 4.5 [3,9]; 3 |
| 9 | 4.5 [1,8];2 | 3.5 [0.5,8]; 4 | 2 [1,4]; 1 | 2 [0.5,3]; 1 | 3.5 [1,4]; 1 | 1.5 [1,3]; 1 | 2 [0.5,2]; 1 |
|  |  |  |  |  |  |  |  |
| patient |  | Oxaliplatin 5 µM | Oxaliplatin 20 µM | Cetuximab 20 nM | Cetuximab 200 nM | Pembrolizumab 140 nM | Pembrolizumab 1400 nM |
| 1 | control vs.: | p > 0.05 | p > 0.05 | p > 0.05 | p > 0.05 | p > 0.05 | p > 0.05 |
| 2 |  | p > 0.05 | p > 0.05 | p > 0.05 | p > 0.05 | p > 0.05 | p > 0.05 |
| 3 |  | p > 0.05 | p > 0.05 | p > 0.05 | p > 0.05 | p > 0.05 | p > 0.05 |
| 4 |  | p = 0.0227 | p > 0.05 | p > 0.05 | p > 0.05 | p > 0.05 | p > 0.05 |
| 5 |  | p = 0.0269 | p > 0.05 | p > 0.05 | p > 0.05 | p > 0.05 | p > 0.05 |
| 6 |  | p > 0.05 | p > 0.05 | p > 0.05 | p > 0.05 | p > 0.05 | p > 0.05 |
| 7 |  | p > 0.05 | p > 0.05 | p > 0.05 | p > 0.05 | p > 0.05 | p > 0.05 |
| 8 |  | p > 0.05 | p > 0.05 | p > 0.05 | p > 0.05 | p > 0.05 | p > 0.05 |
| 9 |  | p > 0.05 | p > 0.05 | p > 0.05 | p > 0.05 | p > 0.05 | p > 0.05 |

Supplementary Table 3: Distribution of tumor, stroma and necrosis in relation to total area. Depicted are untreated (control) and treated (Oxaliplatin, Cetuximab, Pembrolizumab) tissue slices. Shown is the median, minimum, maximum and standard deviation (std.dev.) for all patients.

Supplementary Table 4: Evaluation of PD1 immunostain.

| patient | PD1 pos. immune cells | total tumor cells | IC % |
| --- | --- | --- | --- |
| 1 | 36000 | 100000 | 36 |
| 2 | 25000 | 100000 | 25 |
| 3 | 29000 | 100000 | 29 |
| 4 | 20000 | 100000 | 20 |
| 5 | 41000 | 100000 | 41 |
| 6 | 31000 | 100000 | 31 |
| 7 | 35000 | 100000 | 35 |
| 8 | 21000 | 100000 | 21 |
| 9 | 34000 | 100000 | 34 |

Supplementary Table 5: Evaluation of PD-L1 immunostain.

| patient | PD-L1 pos. tumor cells | PD-L1 pos. immune cells | total tumor cells | CPS | TC % | IC % |
| --- | --- | --- | --- | --- | --- | --- |
| 1 | 100 | 50000 | 100000 | 50,1 | 0,1 | 50 |
| 2 | 500 | 23000 | 100000 | 23,5 | 0,5 | 23 |
| 3 | 100 | 13000 | 100000 | 13,1 | 0,1 | 13 |
| 4 | 2500 | 21000 | 100000 | 23,5 | 2,5 | 21 |
| 5 | 500 | 16000 | 100000 | 16,5 | 0,5 | 16 |
| 6 | 200 | 25000 | 100000 | 25,2 | 0,2 | 25 |
| 7 | 0 | 5000 | 100000 | 5 | 0 | 5 |
| 8 | 0 | 6000 | 100000 | 6 | 0 | 6 |
| 9 | 2000 | 51000 | 100000 | 53 | 2 | 51 |

Supplement Table 6: Clinical information of systemic therapy and duration.

|  | **systemic therapy** | **duaration** | **cessation/side effects** |
| --- | --- | --- | --- |
| **Patient 1** | FOLFOXIRI | 09.08.2018 - 05.09.2018 |  |
|  | FOLFOXIR + Panitumumab | 19.09.2018 |  |
| **Patient 2** | radiochemotherapy | before 12.03.2014 |  |
| **Patient 3** | radiochemotherapy | 31.07.2017 - 01.09.2017 |  |
|  | unknown | 27.11.2017 - 04.03.2018 |  |
| **Patient 4** | radiochemotherapy | 01.03.2017 - 01-04.2017 |  |
|  | Capecitabine | until 01.11.2017 |  |
| **Patient 5** | n.a. | n.a. |  |
| **Patient 6** | radiochemotherapy | 01.07.2016 - 01.08.2016 | discontinued due to side effects |
|  | Capecitabin + Avastin | 01.01.01.2018 | discontinued due to side effects |
| **Patient 7** | FOLFOX | before 01.06.2013 |  |
|  | Bevacizumab + Capecitabine | unknown |  |
|  | FOLFOXIRI + Panitumumab | unknown |  |
|  | FOLFOXIRI + Bevacizumab | 01.10.2016 - 01.05.2017 |  |
|  | Bevacizumab + Fluorouracil | since 01.05.2017 |  |
| **Patient 8** | n.a. | n.a. |  |
| **Patient 9** | FOLFOXIRI + Cetuximab | 15.11.2016 - 01.03.2017 |  |
|  | FOLFOX + Bevacizumab | 29.08.2017 - 01.12.2017 |  |
|  | FOLFOX | 22.12.2017 |  |
|  | radiotherapy | 12.02.2018 |  |
|  | FOLFOX + Bevacizumab | 01.05.2018 |  |
|  | FOLFIRI + Aflibercept | 01.10.2018 - 01.01.2019 |  |
|  | Trifluridin + Tipiracil | since 01.01.2019 |  |

FOLFOXIRI = Folinic acid, Fluorouracil, Oxaliplatin, Irinotecan; FOLFOX = Folinic acid, Fluorouracil, Oxaliplatin
