# Supplementary material for: Ex vivo tissue slice culture system to measure drug-response rates of hepatic metastatic colorectal cancer
Source: BMC Cancer. 2019 Nov 1;19:1030. doi: 10.1186/s12885-019-6270-4 (PMC6824140; doi:10.1186/s12885-019-6270-4)
Supplement: Supplementary file 3 — Additional file 3: Figure S2.. Depicted are H&E, EvG, Ki-67 and Casp 3 stained sections of representative treated (Cetuximab, Pembrolizumab and Oxaliplatin) and untreated (control) tissue slices of patient 5. The upper row depicts H&E stained sections, little boxes show a higher magnification to show nuclear detail. The middle row shows EvG-stained sections and Ki-67 immunostain. The lower row shows Casp 3 Immunostain, little boxes show a higher magnification to show nuclear detail. [file 12885_2019_6270_MOESM3_ESM.pptx]

## Slide 1
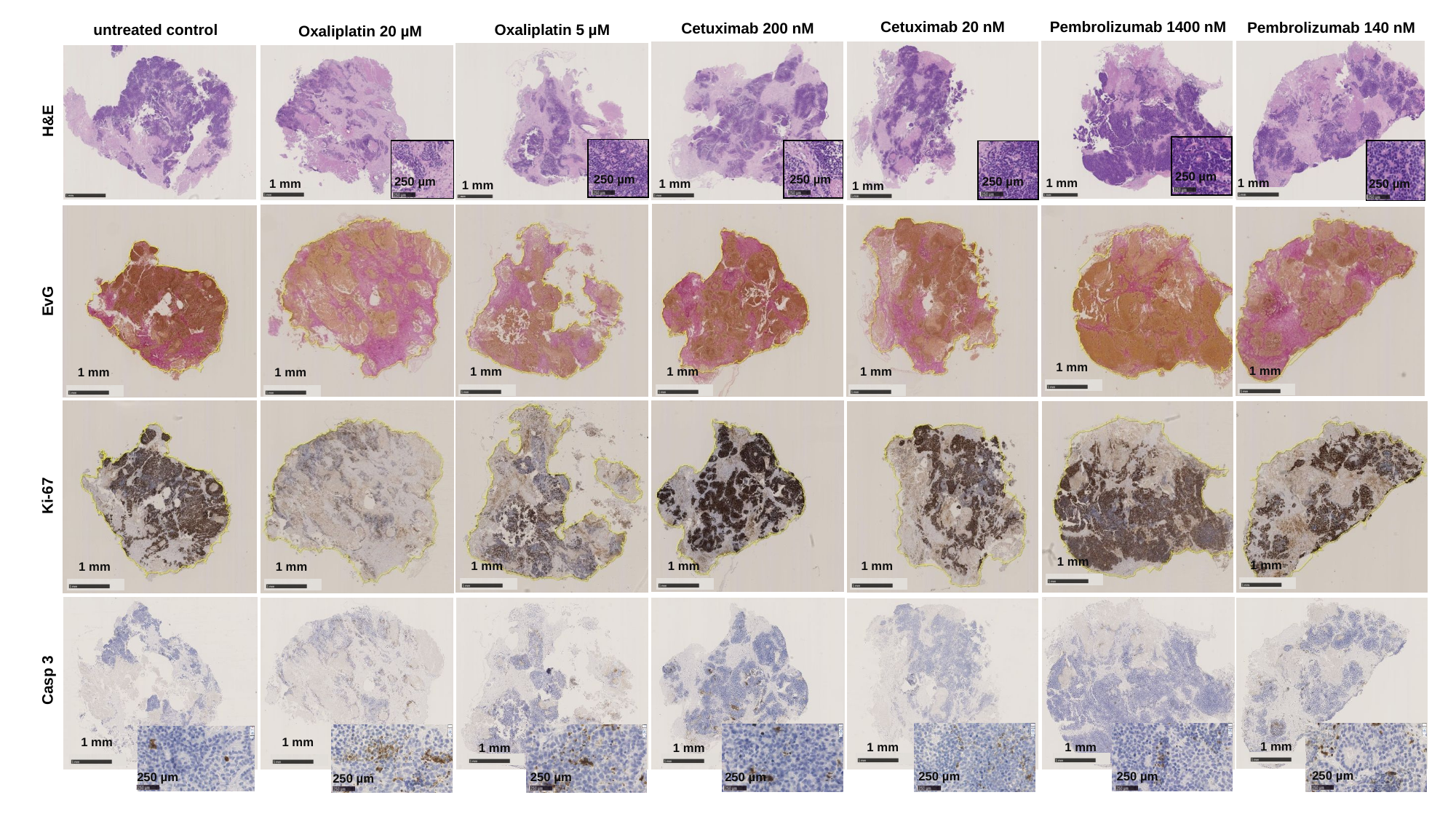

Pembrolizumab 1400 nM
Cetuximab 20 nM
Pembrolizumab 140 nM
Cetuximab 200 nM
untreated control
Oxaliplatin 5 µM
Oxaliplatin 20 µM
H&E
250 µm
250 µm
250 µm
250 µm
250 µm
1 mm
1 mm
1 mm
1 mm
250 µm
1 mm
1 mm
EvG
1 mm
1 mm
1 mm
1 mm
1 mm
1 mm
1 mm
Ki-67
1 mm
1 mm
1 mm
1 mm
1 mm
1 mm
1 mm
Casp 3
1 mm
1 mm
1 mm
250 µm
1 mm
250 µm
1 mm
250 µm
1 mm
250 µm
1 mm
250 µm
250 µm
250 µm
