# Supplementary material for: Ex vivo tissue slice culture system to measure drug-response rates of hepatic metastatic colorectal cancer
Source: BMC Cancer. 2019 Nov 1;19:1030. doi: 10.1186/s12885-019-6270-4 (PMC6824140; doi:10.1186/s12885-019-6270-4)
Supplement: Supplementary file 4 — Additional file 4: Adaptations to Tumor Tissue Slice Culture for Hepatic Colorectal Metastases. More detailed information of the protocol of tumor tissue slice culture is provided. [file 12885_2019_6270_MOESM4_ESM.docx]

**Adaptations to Tumor Tissue Slice Culture for Hepatic Colorectal Metastases**

Hepatic colorectal metastasectomy specimens are a major challenge for the tissue slice culture technique. Vast necrotic areas and chronic fibrous-inflammatory regressive changes due to insufficient supply of blood and nutrients or presurgical therapy render it difficult to sample adequate tissue for the cultivation procedure. Success rates for such an uncontrolled approach were as low as 20% in our experience (unpublished data). In this study, we therefore analyzed a small disc from one end of the tumor-punch and confirmed adequacy by means of frozen section, which was performed in less than 10 min. This approach led to a sufficient tumor-quantity and quality in 97% of all tissue slices and must be recommended for future studies.

For the generation of homogenous tumor tissue slices the Vibratome VT1200 was used that has recently been reported to show the highest accuracy and reproducibility in comparison to other tissue slicers (Vibrocheck and Krumdieck tissue slicer) ^1^. In order to define optimal cultivation criteria, different parameters had been tested before the study. Two metastasectomy specimens were prepared for tissue slice culture and morphologically assessed by three pathologists (WR, DW, SZM, data not shown). The best preservation of tumor morphology was achieved when tissue slices were put on special cell-culture inserts (versus free floating) to preserve the 3-dimensional structure and to assure the supply with oxygen and cell medium by localizing the tissue slice at the air-medium border. This is in line with findings of Vaira et al. and Sönnichsen et al. that also used such cell-culture inserts to cultivate tissue from colorectal carcinoma primaries ^2, 3^. To our knowledge, the only other group to use tissue of primary colon carcinoma for tissue slice culture was Majumder et al. ^4^. They used a more elaborate cultivation technique, where slices were put on flat bottom plates coated with tumor-stromal matrix proteins. Additionally they added autologous patient serum to the cell media. While being described as superior, this technique is very laborious, time- and cost-consuming. In our opinion a successful and quick development of a predictive ex vivo test-system necessitates a widely and easy to reproduce experimental set-up. Therefore, we have not investigated this technique further in this study.

References

**1.** Zimmermann M, Lampe J, Lange S, et al. Improved reproducibility in preparing precision-cut liver tissue slices. *Cytotechnology.* 2009;61:145-152.

**2.** Vaira V, Fedele G, Pyne S, et al. Preclinical model of organotypic culture for pharmacodynamic profiling of human tumors. *Proc Natl Acad Sci U S A.* 2010;107:8352-8356.

**3.** Sonnichsen R, Hennig L, Blaschke V, et al. Individual Susceptibility Analysis Using Patient-derived Slice Cultures of Colorectal Carcinoma. *Clin Colorectal Cancer.* 2018;17:e189-e199.

**4.** Majumder B, Baraneedharan U, Thiyagarajan S, et al. Predicting clinical response to anticancer drugs using an ex vivo platform that captures tumour heterogeneity. *Nat Commun.* 2015;6:6169.
